# The La Crosse virus Gc head domain is a major determinant of infection and pathogenesis

**DOI:** 10.1128/jvi.00892-25

**Published:** 2025-10-31

**Authors:** Ariana Dedvukaj, Nicole C. Rondeau, Tamara J. B. Vázquez, Alejandro E. Cristófalo, Molly V. Durawa, Matthew C. Lutchko, Kenneth A. Stapleford

**Affiliations:** 1 Department of Microbiology, New York University Grossman School of Medicine 12296, New York, New York, USA; 2 Centro de Investigaciones en Bionanociencias (CIBON), Consejo Nacional de Investigaciones Científicas y Técnicas (CONICET) 62873, Buenos Aires, Argentina; University of Kentucky College of Medicine, Lexington, Kentucky, USA

**Keywords:** orthobunyavirus, La Crosse virus, head domain, virus entry

## Abstract

How orthobunyaviruses establish infections and disseminate to cause disease is not well understood. In a previous study using the *in vivo* evolution of La Crosse virus (LACV), we discovered a cluster of mutations localizing to the LACV Gc head domain. However, we do not understand how the Gc head domain contributes to infection. Here, we generated each of the aforementioned mutations and addressed the role of the Gc head domain in viral replication and infectivity in mammalian and insect cells. We found that specific head domain residues could attenuate replication and infectivity in human neurons and reduce cell binding across different hosts, indicating an important role for the head domain during infection *in vitro*. Focusing on the *in vitro*-attenuated Gc N609D variant, we infected 3-week-old wild-type (WT) C57BL/6J mice via the footpad with WT LACV or the Gc N609D variant and found that the Gc N609D virus was completely attenuated. To address whether the variant was also attenuated in a highly susceptible mouse model, we infected *Ifnar1*^−/−^ mice with WT LACV and Gc N609D. We found that virulence in mice infected with Gc N609D was delayed, with several mice surviving the infection, and while viral titers were comparable between viruses in the footpad, there was a significant reduction in virus in the brain. Together, these studies define a critical role of the Gc head domain in infectivity and pathogenesis. Studies are underway to further define how the orthobunyavirus Gc head domain contributes to infection and disease.

**IMPORTANCE:**

Orthobunyaviruses are emerging arboviruses capable of severe disease and explosive outbreaks. However, our understanding of how orthobunyaviruses establish infections or cause disease is not completely understood. The orthobunyavirus Gc glycoprotein contains a variable amino-terminal head domain that forms the tip of the virion trimeric spike, yet it is unclear how the head domain contributes to infection or pathogenesis. In this study, we use LACV and a panel of Gc head domain variants to address the role of the head domain in LACV biology. We found that critical head domain regions are important for virus infectivity and pathogenesis in mice, highlighting an important role for the Gc head domain in orthobunyavirus infection and disease.

## INTRODUCTION

The orthobunyavirus genus (*Peribunyaviridae*) includes a long list of significant human pathogens (1, 2). These arthropod-borne viruses (arboviruses) are transmitted to humans primarily by mosquitoes, midges, and ticks. Recent outbreaks of Oropouche virus (OROV) in Cuba (3) and South America (4–7), along with the prevalence of La Crosse virus (LACV) in the United States, emphasize the clinical relevance of orthobunyaviruses. LACV is mainly found in the East North Central and Appalachian regions of the United States (8). As a member of the California serogroup of orthobunyaviruses, LACV is related to other neuroinvasive human viruses found worldwide, including Jamestown Canyon virus, Inkoo virus, and Tahyna virus (9). Although the majority of cases are asymptomatic and therefore underreported, LACV is the leading cause of pediatric arboviral encephalitis in the United States. Neuroinvasive disease can result in fatality or lifelong neurological sequelae, such as recurring seizures and cognitive deficits (10–12). However, there are currently no antiviral therapies or vaccines targeting orthobunyaviruses, highlighting the need to study orthobunyavirus biology in molecular detail.

Our lack of antiviral therapies is in part due to our incomplete understanding of the molecular mechanisms orthobunyaviruses use to establish infections. The orthobunyavirus negative-sense RNA genome consists of S, M, and L segments. The S segment encodes the nucleoprotein and interferon antagonist non-structural protein NSs (13, 14). The M segment encodes the Gn and Gc glycoproteins, along with non-structural protein NSm (1, 15), and the L segment encodes the RNA-dependent RNA polymerase. The M segment proteins are important for virion assembly (15, 16), entry and attachment (17–19), and cell-to-cell spread (20), yet we know little of how discrete domains within these proteins contribute to virus infection. Specifically, the orthobunyavirus Gc protein is a class II fusion glycoprotein similar to those of other bunyaviruses as well as alpha- and flaviviruses (21–24). Orthobunyavirus Gc contains a unique amino-terminal variable head domain that forms the tip of the glycoprotein spike (25, 26). Previous work with Bunyamwera virus and OROV has shown that the head domain can be deleted with little impact to virus replication *in vitro* and that fusion and immunogenicity are enhanced, suggesting that the head domain plays an important yet unknown role in virus biology (27, 28). However, even considering observations, we do not understand specifically how the Gc head domain contributes to infection or pathogenesis.

In a previous study, we hypothesized that we could use *in vivo* virus evolution to identify critical determinants of LACV infection as we have done previously for CHIKV (29, 30) and ZIKV (31, 32). We infected *Aedes (Ae*.) mosquitoes and suckling mice with wild-type (WT) LACV and Sanger sequenced virus populations at 7 and 3 days post-infection for mosquitoes and mice, respectively (17). From these studies, we identified seven mutations in the LACV Gc head domain, suggesting that the head domain may be an important determinant for LACV biology (17). Here, we generated this panel of LACV Gc head domain variants and addressed the role of these residues and the Gc head domain *in vitro* and *in vivo*. We found that specific Gc head domain residues could influence replication, infectivity, and binding *in vitro*. In addition, we found that the most attenuated Gc variant N609D could completely attenuate virulence of a pathogenic WT LACV strain in wild-type mice. Moreover, we found that Gc residue N609 was critical for infection and virulence in *Ifnar1*^*−/*−^ mice. Finally, using an evolutionary approach, we found that the LACV Gc head domain has been evolving across LACV lineages and that there are conserved elements maintained within the head domain of related orthobunyaviruses. Together, these studies provide a critical role for the orthobunyavirus Gc head domain in virus binding, infectivity, and virulence and allow us to speculate on how changes in the orthobunyavirus head domain may impact virus biology.

## RESULTS

### LACV Gc head domain variants localize to the tip of the head domain with the potential to alter folding and trimer stability

**Fig 1:**
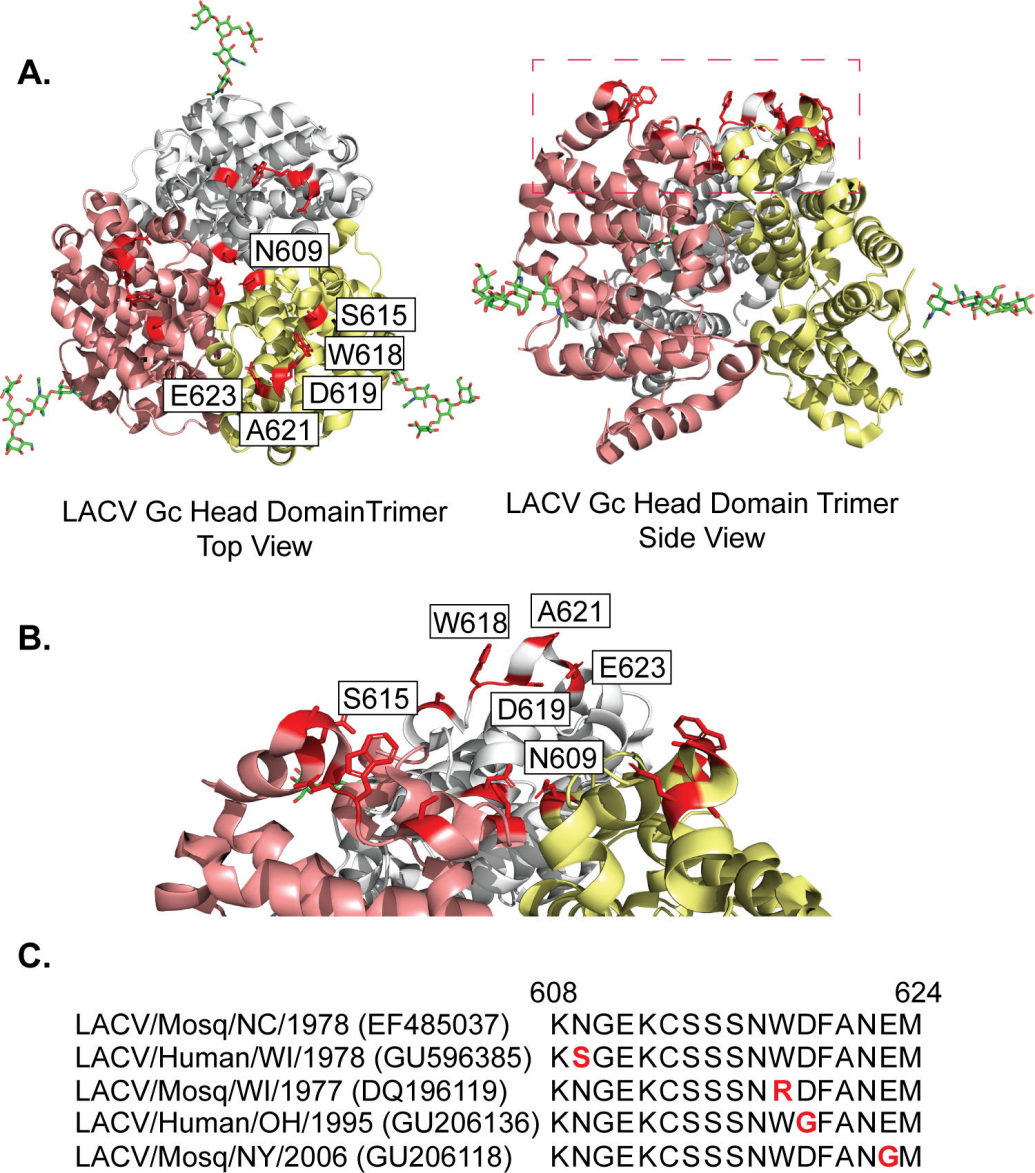
La Crosse virus (LACV) Gc head domain trimer localization. (**A**). Crystal structure of the LACV Gc head domain trimer (PBD: 6H3W) top and side view with variants shown in red. The red dashed box corresponds to panel B. (**B**) Zoom-in view of the top of the LACV Gc head domain trimer outlined by the dashed red box in panel A. (**C**) LACV sequence alignments of the Gc head domain variant amino acid regions 608 to 624. Accession numbers are located to the right of each name. Natural variants are shown in red.

The orthobunyavirus Gc glycoprotein amino-terminal variable head domain sits atop the glycoprotein spike (Fig. 1A and B, red dashed box) (24–26). However, it is unclear how the head domain contributes to LACV infection *in vitro* or *in vivo*. In a previous study, we used *in vivo* evolution of LACV in *Ae*. mosquitoes and C57BL/6J mice to identify potential residues that may be important for virus infection (17). We identified seven variants at six head domain residues, which we hypothesized were important for LACV infection (Table 1; Fig. 1).

**TABLE 1:** LACV Gc head domain variant folding and binding free energy values

Variant^*a*^	ΔΔ_fold_G (kcal/mol)^*b*^	ΔΔ_bind_G (kcal/mol)^*b*^
N609D^Mosq^	13 ± 2	8 ± 2
S615I^Mosq^	2.9 ± 0.5	−0.02 ± 0.04
W618R^Mosq/Mice^	0.5 ± 0.6	−0.009 ± 0.002
W618L^Mosq^	1.3 ± 0.1	−0.001 ± 0.001
D619G^Mosq/Mice^	11.4 ± 0.2	0.0007 ± 0.0002
A621V^Mice^	6.9 ± 0.4	0.0003 ± 0.0003
E623A^Mosq^	1.3 ± 0.4	−0.151 ± 0.002

^*a*^ Mosq, found in mosquitoes; Mice, found in mice.

^*b*^ Positive values imply negative impacts on trimer folding or inter-protomer binding.

To understand how these residues contribute to LACV biology, we first looked at where each residue is located on the Gc head domain trimer (Fig. 1A through C) (17, 26). We observed that residue N609 is positioned at the point at which all three monomers converge to form the trimer, while residues S615, W618, D619, A621, and E623 are positioned above N609 on extensions facing away from the particle (Fig. 1A through C). The location of these residues, particularly N609, allowed us to hypothesize that changes at these amino acids may impact trimer folding. To test this hypothesis *in silico*, we calculated the ΔΔ_fold_G and ΔΔ_bind_G for each variant to understand how individual variants may influence trimer folding and monomer binding, respectively (Table 1). We observed that the variant N609D is predicted to negatively impact trimer folding and inter-protomer binding, while other variants such as D619G and A621V are predicted to have impacts on trimer folding but not inter-protomer interactions. Finally, we addressed whether mutations at the residues in Table 1 have been observed in nature. We aligned the M segment protein regions deposited in the National Center for Biotechnology and Information (NCBI) Virus and found several of the variants we identified (W618R, D619G, and E623G) have been found in nature (Fig. 1C). Taken together, these studies highlight the potential role of the Gc head domain in LACV infection.

### LACV Gc head domain is important for virus production in human cells

**Fig 2:**
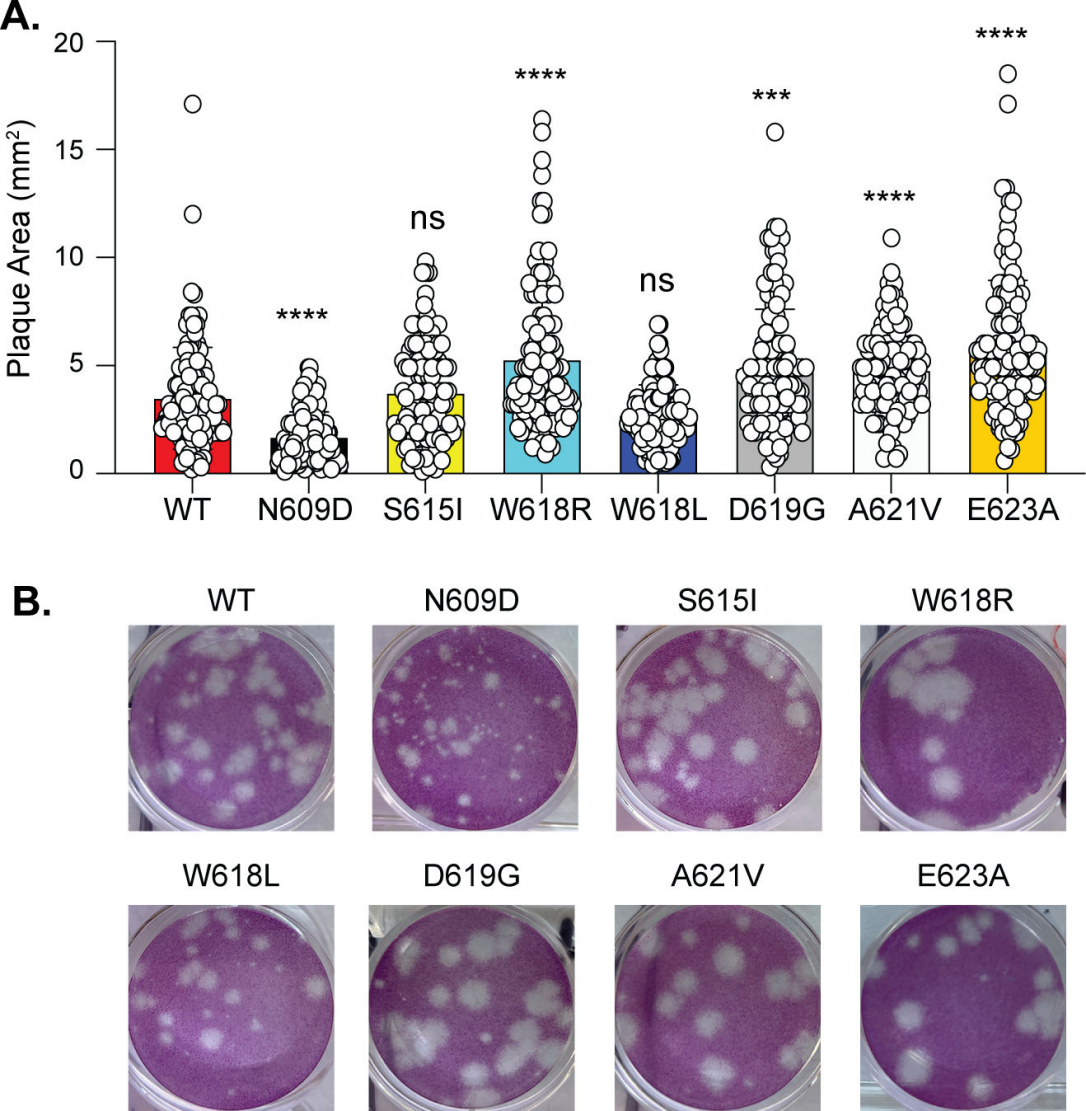
LACV Gc head domain variants influence plaque size. (**A**). Plaque size of wild-type LACV and each Gc head domain variant on Vero cells 72 h post-infection. Results represent the mean and SD of two independent infections. *N* = 120 plaques for each virus. Kruskal-Wallis test. ***P < 0.001, ****P < 0.0001. ns, non-significant. (**B**) Represented images of plaques of each virus.

Given the location of each residue and the potential influence of each variant on folding, we hypothesized that the head domain is critical for virus infection. To test this hypothesis, we generated each head domain variant in the LACV lineage I Mosquito/NC/1978 infectious clone M segment (33). All variants were genetically stable after passaging virus once in Vero cells to generate working stocks. To begin, we looked at plaque size of each variant on Vero cells (Fig. 2). We observed that the variants Gc N609D and W618L led to small plaques, while variants W618R, D619G, A621V, and E623A led to larger plaques compared to WT LACV (Fig. 2).

**Fig 3:**
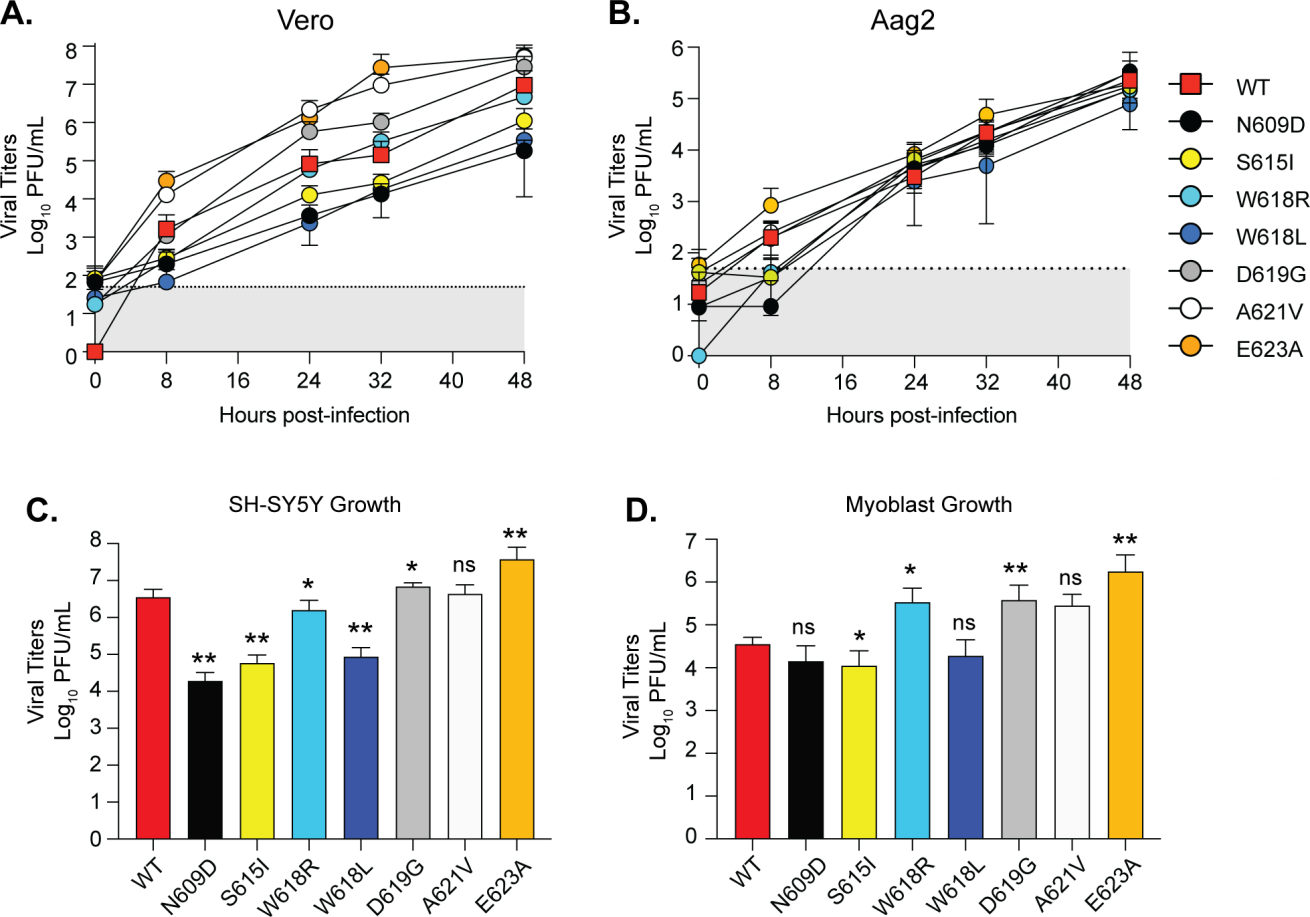
LACV Gc head domain growth in mammalian and insect cells. Vero (**A**), Aag2 (**B**), human SH-SY5Y (**C**), and human myoblast (**D**) cells were incubated with each virus at an MOI of 0.1 for 1 h at 37°C. After incubation, cells were washed, and complete media were added to each well. The supernatant was collected at the indicated time point, and infectious virus was quantified by plaque assay on Vero cells. SH-SY5Y and myoblasts were harvested at 24 h post-infection. Data represent the mean and SD. Three independent experiments, *N* = 6 for each virus. Mann-Whitney test. *P < 0.05, **P < 0.01. ns, non-significant.

To study the role of each residue in LACV replication *in vitro*, we first performed multi-step growth curves by infecting Vero cells with each virus at a multiplicity of infection (MOI) of 0.1 and quantifying infectious virus in the supernatant at multiple time points post infection (Fig. 3A). Vero cells were used because of their interferon signaling deficiency, allowing us to focus on the role of each Gc residue without confounding influence from the antiviral response. In Vero cells, the replication of the Gc variants separated into two groups. We found that several of the variants, including Gc N609D, S615I, and W618L, led to attenuated growth, while D619G, A621V, and E623A led to enhanced growth over WT LACV (Fig. 3A). Since several of the variants were selected for in *Ae. aegypti* mosquitoes, we also addressed viral growth in Aag2 *Ae. aegypti* cells by infecting Aag2 cells with each virus at an MOI of 0.1 and quantifying infectious virus in the supernatant by plaque assay (Fig. 3B). We observed that while several of the variants were attenuated early during infection, all of the variants caught up to WT LACV by 24 h. When we sequenced each variant at 24 h from the Aag2 cells, we found that all of the variants had reverted to the wild-type residue, suggesting that these variants are restricted early during infection, but a strong selective pressure forces them to revert to the WT residue. Together, given significant changes in Vero cells and reversion in Aag2 cells, these studies show that the Gc head domain plays an important role in replication *in vitro*.

We next tested whether the LACV Gc head domain residues were critical for replication in human neurons and myoblasts, relevant cell lines for LACV infection (34, 35) (Fig. 3C and D). We infected human neuroblastoma SH-SY5Y cells and human myoblasts with WT LACV or each Gc head domain variant at an MOI of 0.1 and quantified infectious virus production at 24 h post-infection. In SH-SY5Y cells, we found that the variants Gc N609D, S615I, and W618L were attenuated in virus growth up to 100-fold compared to WT LACV, similar to the growth in Vero cells (Fig. 3A). In human myoblasts, we observed that the attenuated Gc N609D, S615I, and W618L variants in neurons and Vero cells were not as severely impacted. On the other hand, we found that the Gc variants W618R, D619G, A621V, and E623A were able to enhance infection, similar to what we saw in Vero cells. These results demonstrate that the Gc head domain can influence virus production in multiple cell types, including human cells, and that there may be cell type-specific mechanisms regulating infection.

### The LACV Gc head domain residues contribute to cell-specific infectivity and binding

**Fig 4:**
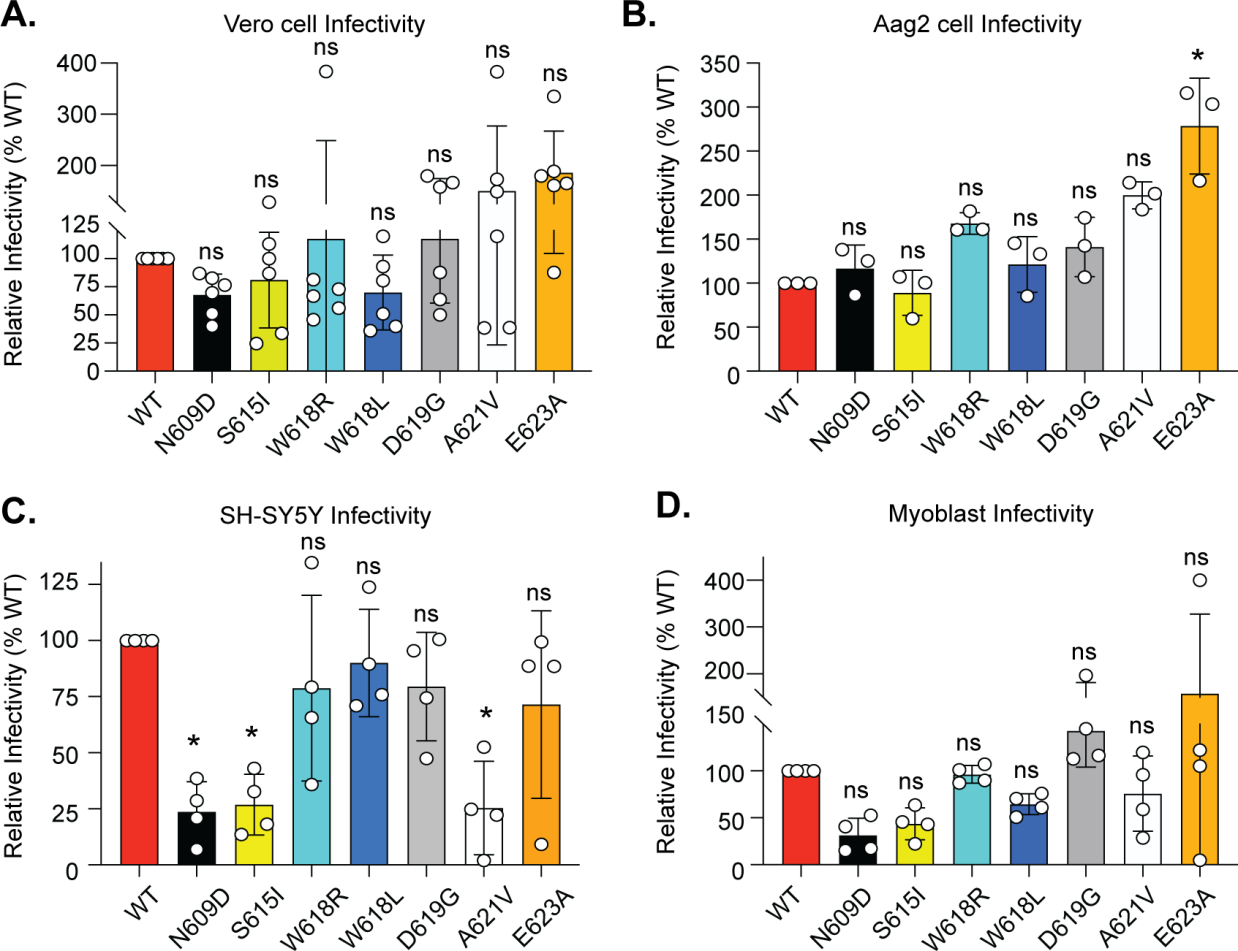
LACV Gc head domain infectivity in multiple cell types. Vero cells (**A**), Aag2 cells (**B**), human SH-SY5Y (**C**), or myoblasts (**D**) were incubated with each virus at an MOI of 1 for 1 h at 37°C. Following incubation, complete media containing 20 mM NH_4_Cl were added, and the cells were incubated for 24 h at 37°C. Cells were then fixed, stained with LACV antisera and 4′,6-diamidino-2-phenylindole (DAPI), and the number of infected cells was quantified by microscopy. Data are normalized to WT LACV infection. Data represent the mean and SD of three to six independent experiments. Kruskal-Wallis test. *P < 0.05. ns, non-significant.

Given the location of each residue on the Gc spike (Fig. 1) and our plaque size phenotypes (Fig. 2), we hypothesized these residues may be important for virus infectivity. To test this hypothesis, we performed infectivity assays by incubating WT LACV and each variant with Vero cells, Aag2 cells, human neurons, or myoblasts at an MOI of 1 for 1 h to allow entry. We then added media containing 20 mM ammonium chloride (NH_4_Cl) to stop further virus spread, allowing us to address only the initial infection. In Vero cells (Fig. 4A) and Aag2 cells (Fig. 4B), we observed that many of the variants had little impact on virus infectivity. However, the LACV variant E623A led to enhanced infection in both Vero and Aag2 cells, suggesting a role in entry. On the other hand, in both human neurons (Fig. 4C) and myoblasts (Fig. 4D), we found that the Gc variant N609D and S615I had reduced virus infection, indicating that these variants may have defects in virus entry. Interestingly, the Gc variant A621V was also attenuated in the neurons (Fig. 4C) yet showed the same levels of infectious particle production in growth assays as WT LACV (Fig. 3A). These results may suggest that the Gc A621V variant could have defective cell entry, rescued by advantages in virus genome replication, assembly, or egress.

**Fig 5:**
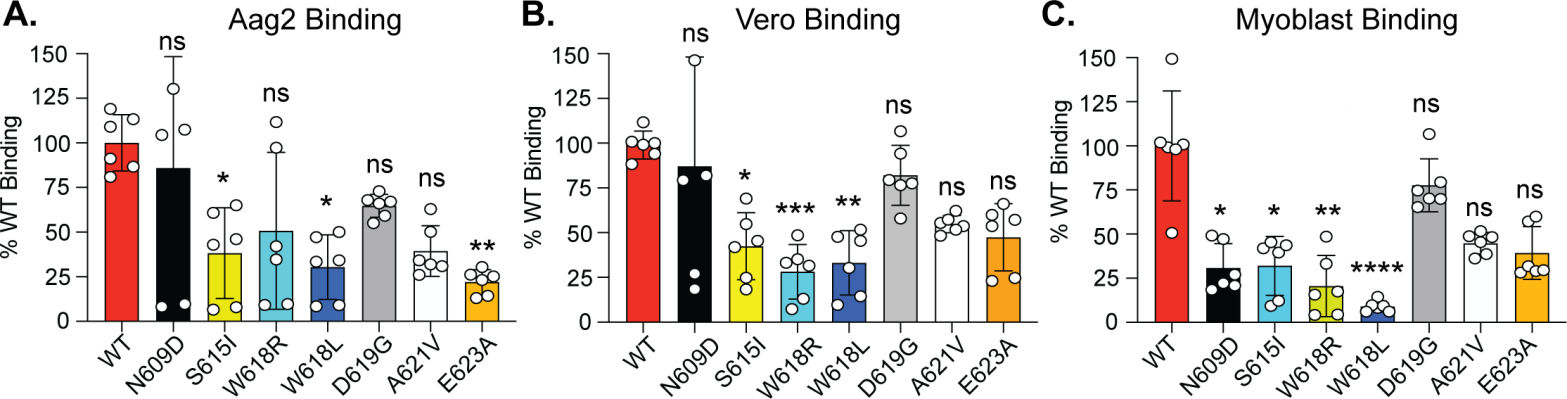
Cell binding of LACV Gc head domain variants. Aag2 cells (**A**), Vero cells (**B**), or human myoblasts (**C**) were treated with 20 mM NH_4_Cl for 1 h and then placed on ice. Cells were incubated with each virus at an MOI of 100 (based on S segment RNA genomes) for 30 min on ice. Cells were washed extensively; RNA was extracted; and relative S segment and actin (Aag2) or GAPDH (Vero and myoblasts) RNA levels were quantified by qPCR. Relative binding was determined using the 2^−ΔΔCT^ methods and represented as a percentage of WT virus binding. Data represent the mean and SD of three independent experiments (*N* = 6). Kruskal-Wallis test. *P < 0.5, **P < 0.01, ***P < 0.001, ****P < 0.0001. ns, non-significant.

To begin to understand how the Gc head domain functions during entry, we performed cell binding assays on Vero, Aag2, and human myoblasts (Fig. 5). We chose to omit the human neurons as they did not adhere to culture plates well enough to withstand vigorous washing. We incubated each cell line with 20 mM NH_4_Cl for 1 h to block endocytosis and then added sucrose-purified viruses (MOI of 100 based on RNA molecules) for 30 min on ice. We then washed off unbound virus and quantified the amount of bound virus S segment RNA by qPCR. We found that the Gc residues S615, W618, and E623 could alter binding in all three cell lines, while residue D619 behaved similarly to WT virus. Interestingly, residue N609 did not play a major role in binding in Vero or Aag2 cells (Fig. 5A and B) but did influence binding in myoblasts (Fig. 5C), suggesting cell type-specific roles in virus infection as we saw for infectivity. Taken together, we conclude that the LACV Gc head domain is critical for virus entry and cell binding.

### The Gc head domain variant N609D attenuates LACV virulence and replication in mice

**Fig 6:**
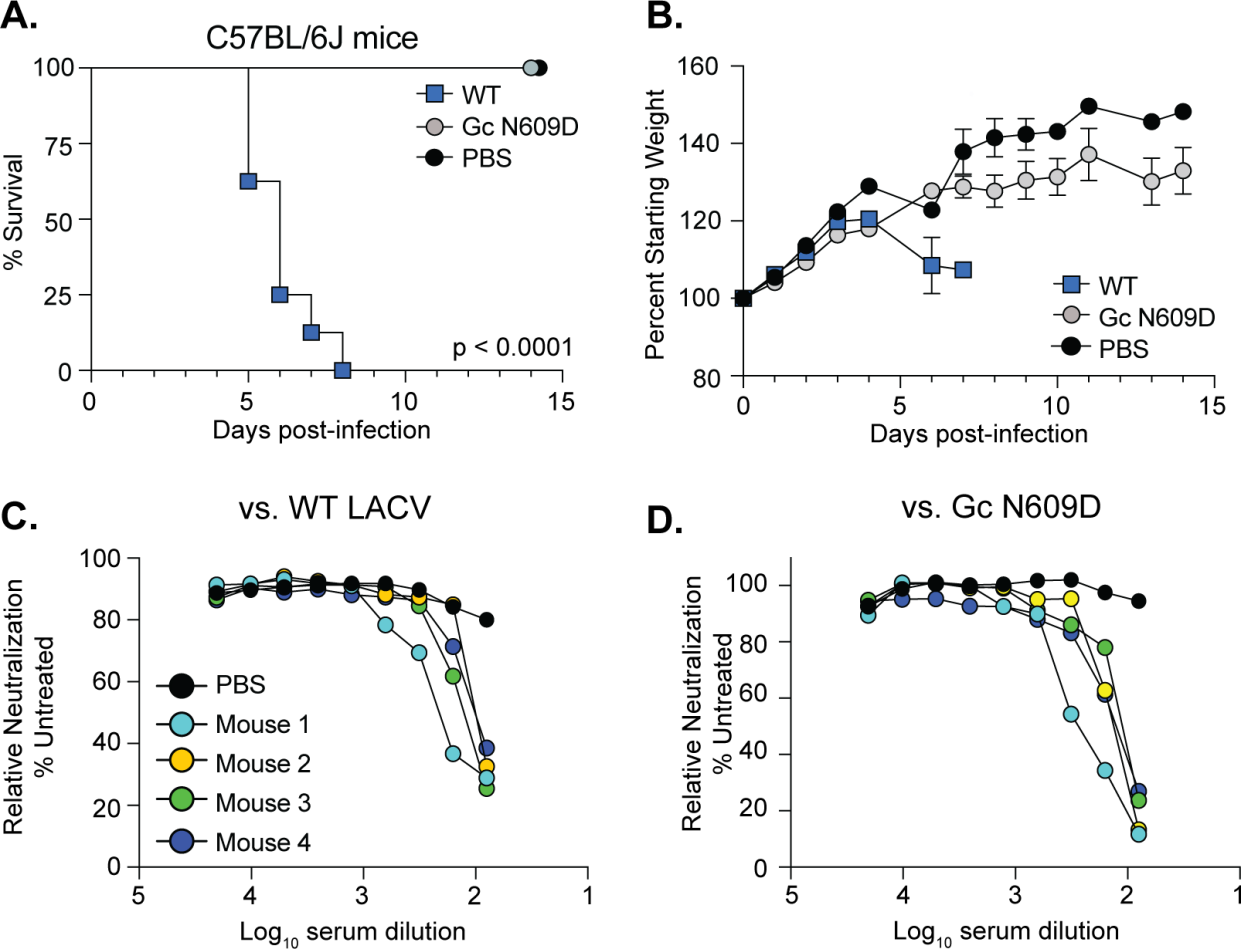
The Gc variant N609D is attenuated in wild-type mice. (**A and B**) Three-week-old male and female C57BL/6 J mice were infected with 20,000 PFU of each virus or phosphate-buffered saline (PBS) via the footpad. Mice were monitored (**A**) and weighed daily (**B**). Data represent two independent infections. *N* = 8 mice for each virus. Mantel-Cox test. (**C and D**) At 14 days post-infection, surviving mice were euthanized, and the serum was collected to address neutralizing antibodies. Serum was inactivated, and twofold dilutions were mixed with 5,000 PFU of WT LACV (**C**) or the Gc N609D virus (**D**) for 1 h at 37°C. Following incubation, the virus-serum mix was added to Vero cells for 48 h. Cells were fixed and stained, and the number of infected cells was quantified by microscopy.

The Gc head domain variant N609D displayed the strongest phenotypes *in vitro* with reduced replication and infectivity in neurons, the major site of infection in humans and mice. These results allowed us to hypothesize that the Gc N609D variant may significantly reduce virulence. To test this hypothesis, we infected 3-week-old wild-type C57BL/6J mice with 20,000 PFU of WT LACV or the Gc N609D via the left footpad and weighed the mice daily for 2 weeks (Fig. 6A and B). We found that while all of the mice infected with WT LACV succumbed to infection by 8 days post-infection, the mice infected with Gc N609D all survived the infection (Fig. 6A). One potential explanation for this phenotype is that the mice injected with the N609D variant did not become infected. To rule this possibility out, we addressed the presence of neutralizing antibodies in each mouse and found that the mice infected with Gc N609D generated antibodies that neutralized both WT LACV and the Gc N609D variant (Fig. 6C and D), indicating that they were infected.

**Fig 7:**
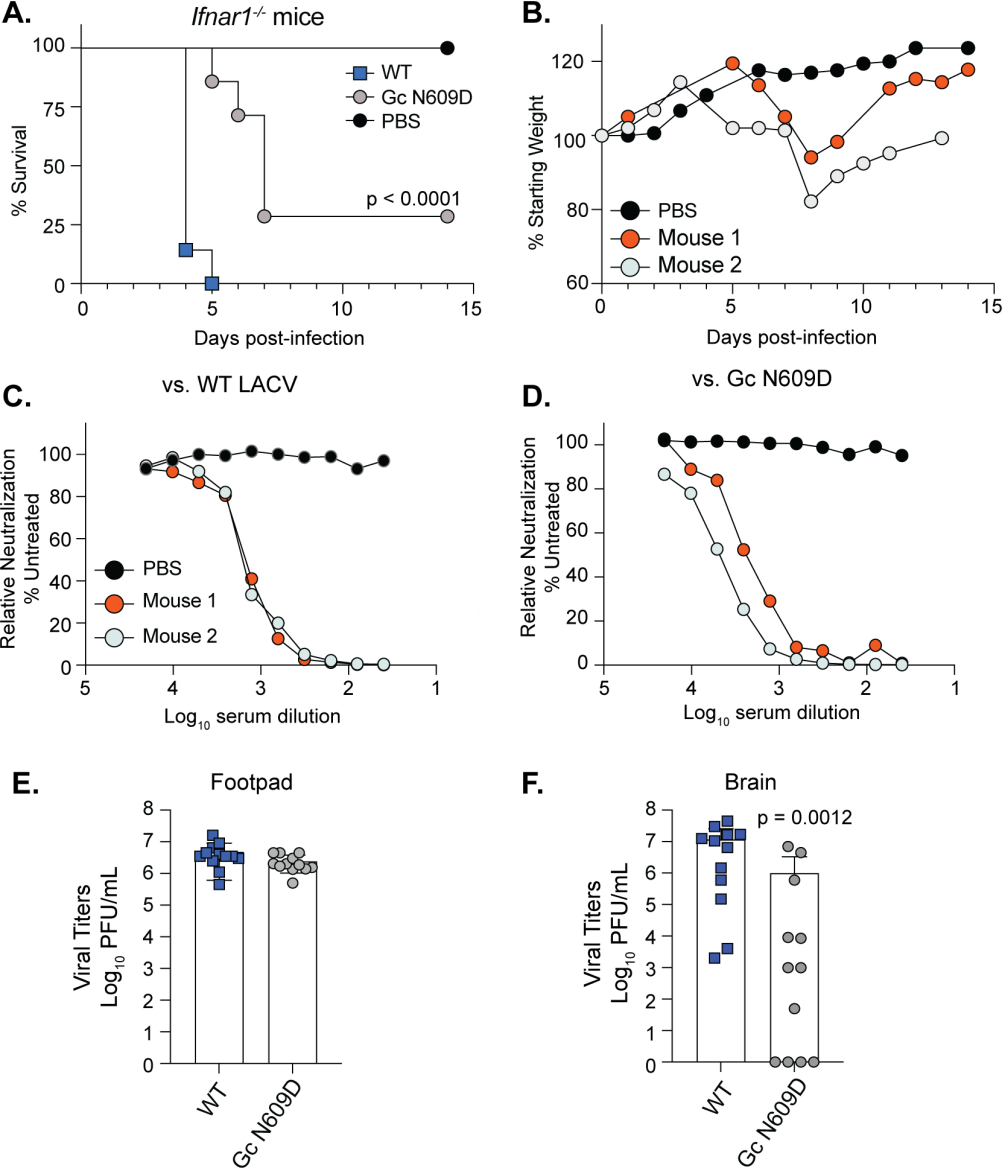
The Gc head domain is important for virulence and infection in *Ifnar1*^−/−^. (**A and B**) Three-week-old male and female *Ifnar1^−/−^* mice were infected with 50,000 PFU of each virus or PBS via the footpad. Mice were monitored (**A**) and weighed daily (**B**). Data represent two independent infections. *N* = 7 mice for each virus. Mantel-Cox test. (**C and D**) At 14 days post-infection, surviving mice were euthanized, and the serum was collected to address neutralizing antibodies. Serum was inactivated, and twofold dilutions were mixed with 5,000 PFU of WT LACV (**C**) or the Gc N609D virus (**D**) for 1 h at 37°C. Following incubation, the virus-serum mix was added to Vero cells for 48 h. Cells were fixed and stained, and the number of infected cells was quantified by microscopy. (**E and F**) Three-week-old male and female *Ifnar1*^−/−^ mice were infected with 20,000 PFU of each virus or PBS via the footpad. At 3 days post-infection, mice were euthanized, and the footpad (**E**) and brain (**F**) were collected and homogenized, and the infectious virus was quantified by plaque assay. Data represent the mean and SD of two independent experiments. *N* = 12 mice for each virus. Mann-Whitney test.

Given the complete attenuation of the LACV Gc N609D variant in WT mice, we wondered whether this variant would also be attenuated in a more susceptible mouse model. We infected *Ifnar1*^−/−^ mice, which lack the type I interferon alpha receptor, with 50,000 PFU of WT LACV or the Gc N609D variant via the footpad (Fig. 7). We observed that all mice infected with WT LACV succumbed to infection at 5 days post-infection. However, we found that for mice infected with Gc N609D, survival was extended several days, with two out of seven mice surviving the infection (Fig. 7A and B). The mice surviving the infection lost weight yet recovered around day 7 and generated neutralizing antibodies to WT LACV and the Gc N609D variant (Fig. 7C and D).

Finally, we hypothesized that the attenuation of the Gc N609D variant in *Ifnar1*^−/−^ mice was due to reduced infection in the brain. To test this hypothesis, we infected *Ifnar1*^−/−^ mice with 20,000 PFU of WT LACV and the Gc N609D virus and measured viral titers in the footpad (site of infection) and brain at 3 days post-infection. In the footpad (Fig. 7E), both viruses replicated to similar levels, indicating that the Gc N609D variant is capable of infecting cells and replicating at the site of infection. However, when we looked at viral titers in the brain at 3 days post-infection, we found that while WT LACV replicated to high titers, many of the mice infected with the Gc N609D variant had significantly reduced viral titers (Fig. 7F) with several mice having no detectable infectious virus in the brain. These results indicate that the Gc head domain, specifically residue N609, is important for virus infection and pathogenesis.

### The Gc head domain conservation across LACV lineages and other orthobunyaviruses

**Fig 8:**
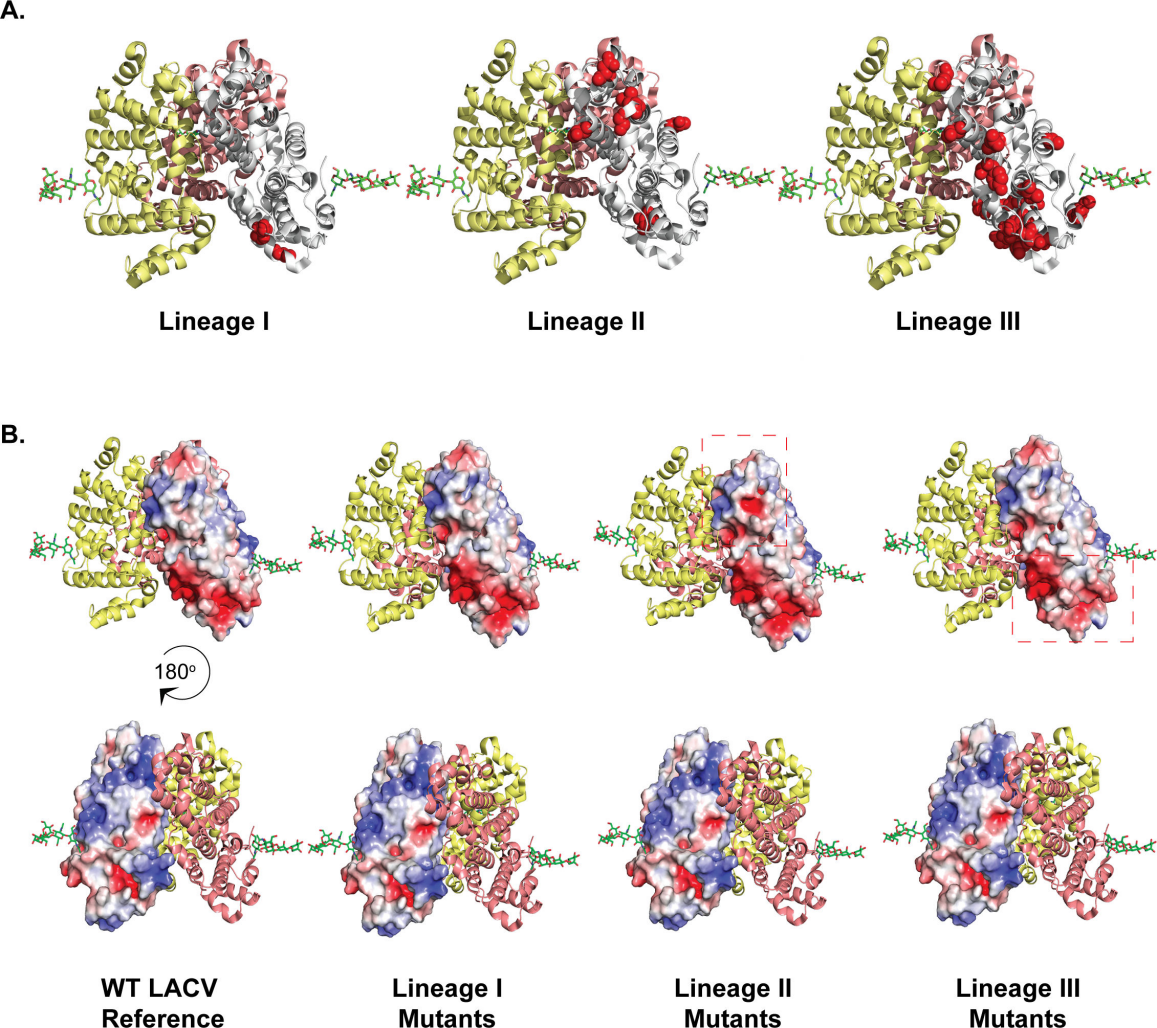
Changes in the Gc head domain across LACV lineages. (**A**) Natural mutations found in the lineage I, II, and III Gc head domains are shown in red. The prototypical LACV/Human/MN/1960 strain was used as a reference, and mutations were considered major changes if they were found in > 60% of the sequences available. (**B**) Electrostatic potentials of the reference LACV head domain trimer (PBD: 6H3W) and the lineage I, II, and III natural Gc head domain variants. Negative electrostatic potential is shown in red, and positive electrostatic potential is shown in blue. Red dashed boxes are regions of charge changes between lineages.

Our *in vivo* evolution studies identified the Gc head domain as a potential hotspot for adaptation (17). Therefore, we asked whether the LACV Gc head domain has been changing over time and between LACV lineages. We aligned the LACV Gc head domain regions (amino acids 477 to 722) of all complete LACV M segment sequences (31 in total) deposited in the NCBI Virus database (Fig. S1). For LACV lineage I, we found that the Gc head domain remained mostly constant with only two mutations present in over 60% of deposited sequences (V528I and K548R; Table 2; Fig. 8A). These two residues are found at the base of the head domain away from the mutations we had found in our *in vivo* study. In LACV lineage II, there were six mutations with five localized to the tip of the head domain (Table 2; Fig. 8A). Finally, in LACV lineage III, which is the most divergent lineage (36), there were 19 amino acid changes that largely localized along the side of the head domain and at the base (Fig. 8A).

**TABLE 2:** LACV Gc head domain mutations across lineages

Lineage I	Lineage II	Lineage III
V528I	I557T	T479S
K548R	D626N	T480S
	T633A	T515A
	T679A	I530M
	S699G	G533E
	G702D/E	G540S
		K548R
		D550A
		I557A
		S563T
		K564R
		L597M
		T633A
		T660A
		A672T
		N705S
		Y708H
		N720T
		A722T
		S724P

Looking closer at the differing residues between lineages, many of the changes in the Gc head domain corresponded to changes in charge, leading us to hypothesize that these may alter the overall charge of the Gc head domain. To address this hypothesis, we first looked at the overall charged distribution of the LACV Gc head domain in the trimer (Fig. 8B). On one side of the Gc head domain, there is a largely negatively charged region running along the side of the head domain with a negatively charged spot on top and a positively charged region on the side (Fig. 8B). When we introduced changes for lineages I, II, and III into the LACV head domain structure, we found that these variants changed the overall charge of the Gc head domain both at the tip, the side, and the base of the head domain (Fig. 8B, boxes). These results suggest that alterations in the Gc head domain can influence charged patches on the protein surface that are critical for protein-protein interactions within the virus or host-pathogen interactions needed for infection.

**Fig 9:**
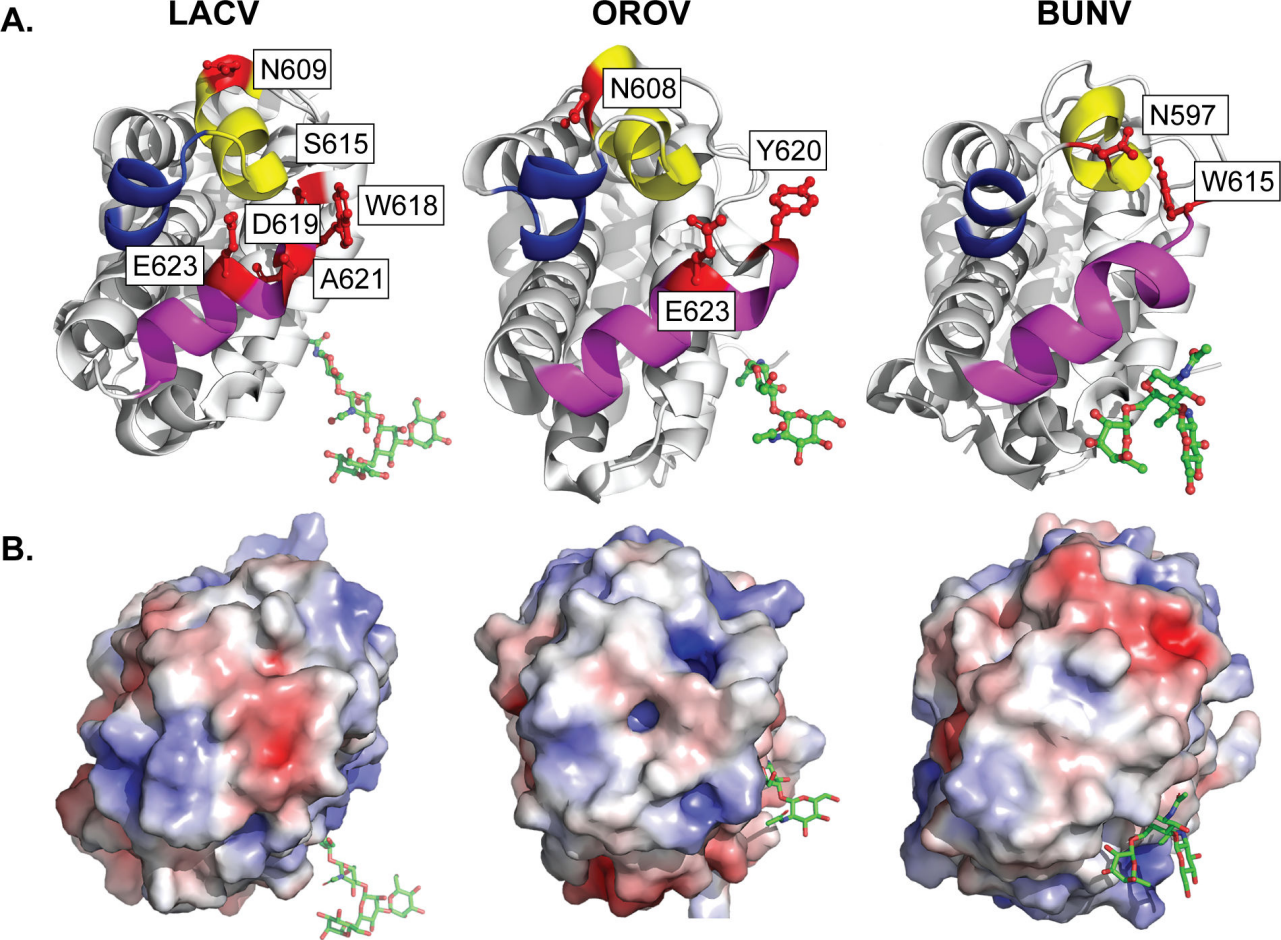
Conservation of the Gc head domain among orthobunyaviruses. (**A**) Crystal structure of the LACV (PDB: 6H3W), OROV (PDB: 6H3X), and BUNV (PDB: 6H3V) Gc head domains with residues shown in red. (**B**) Electrostatic potential of the LACV, OROV, and BUNV Gc head domain tip.

Finally, we asked whether the Gc head domain residues we studied in LACV were conserved across other orthobunyaviruses (Fig. 9). Looking at the head domain of the related orthobunyaviruses OROV and BUNV, we found that there are structurally similar alpha helices making up the top portion of the head domains (Fig. 9A, colored helices). Moreover, there are similar amino acid residues present in the top of the orthobunyavirus head domains, including a conserved glutamine, which we showed in LACV is critical for pathogenesis, as well as a conserved aromatic tryptophan or tyrosine. Interestingly, both LACV and OROV contain an identical glutamic acid at position 623, which we have shown is capable of enhancing LACV infection *in vitro*. While there are similarities between orthobunyavirus Gc head domains at the amino acid level, we found major differences in the charge distribution of the Gc head domain between viruses (Fig. 9B). These results suggest that the location of charged residues could impact critical host-pathogen interactions necessary for individual virus biology.

## DISCUSSION

Orthobunyaviruses are significant human pathogens capable of devastating outbreaks. These viruses encode a class II fusion glycoprotein Gc that is critical for virion assembly and entry. The Gc glycoprotein is functionally and structurally similar to those of alpha- and flaviviruses in domain II, which includes the fusion loop. However, orthobunyaviruses are unique in that they encode a variable amino-terminal head domain that forms the tip of the virion trimeric spike. Our understanding of how the head domain functions is not well defined.

In this study, we took advantage of *in vivo* evolution of LACV in mice and mosquitoes, identifying several mutations clustering in the Gc head domain. Given the location of these changes and that several of them are found in nature, we hypothesized these are important for LACV and orthobunyavirus biology. We generated each variant and tested how these mutations influenced virus growth in multiple cell lines. We found that while these variants were largely genetically stable in mammalian cells, they reverted to the wild-type residue in mosquito cells, suggesting a strong selective pressure in these cells. These results are interesting as we identified several of these variants in mosquitoes. One explanation for these results could be that our previous *in vivo* evolution studies used the LACV lineage I strain Human/MN/1960, which differs from our infectious clone lineage I system Mosquito/NC/1978 by 29 amino acids in the M segment. Regardless, we find that specific head domain residues could influence plaque size, virus production, and infectivity in human neurons and myoblasts, indicating an important role for the head domain in virus production and spread. Interestingly, these specific phenotypes may be cell type specific through our findings of several of the variants being attenuated in neurons but not in myoblasts. Moreover, although we find that the variants W618R, D619G, A621V, and E623A can increase virus production in myoblasts, this does not seem to be due to changes in infectivity. These results may suggest that these variants are important for different steps in the viral life cycle or that these variants enhance other functions to compensate for defects. This point is particularly true when looking at cell binding. We find that many of the variants tested lead to reduced cell binding on multiple cell types, yet these results do not directly translate to similar defects in infectivity. Future studies to explore the detailed mechanisms of how the LACV Gc head domain contributes to fusion and entry will be critical to dissect this complex process. Taken together, our results show that even single amino acid changes can have major impacts on the virus life cycle, highlighting the importance of the Gc head domain in orthobunyavirus biology.

In addition to roles *in vitro*, we found that the head domain and specifically residue N609 were important for virulence in mice. In WT mice, the Gc head domain variant N609D was completely attenuated, yet the mice started to lose weight around 7 days post-infection and retained this weight for the remainder of the experiment. These results, along with the presence of neutralizing antibodies, show that the mice are infected yet do not succumb to infection. Moreover, we find that in the highly susceptible type I interferon-deficient mice, the Gc N609D variant is attenuated, although mice do succumb to infection. In the two *Ifnar1*^−/−^ mice that survived the infection, both lost weight and recovered and produced neutralizing antibodies, which we hypothesize are critical for viral clearance. Looking at virus replication, we find that while the Gc N609D variant can replicate to WT levels at the site of infection, there was a reduction in viral titers in the brain of the same animals at 3 days post-infection. One explanation for these results could be that the Gc head domain is important for neuroinvasion and infection of the brain, as we do see reduced replication and infection in human neurons. Another possibility could be that the Gc head domain is important for virus dissemination from the site of infection to the brain. Similar defects in dissemination have been seen for LACV fusion loop mutants (37); however, these variants also showed replication defects in the muscle, suggesting a global defect in virus entry. Future work investigating multiple time points and organs will be important to understand how the Gc head domain influences infection *in vivo*. Our *in vivo* and *in vitro* results suggest that the Gc head domain is critical for cell-specific interactions and entry mechanisms that may drive virus dissemination. Together, these results show that the Gc head domain plays an essential role in LACV virulence and suggest that if the virus can be restricted to the periphery for long enough that antibodies can be produced, the host can recover.

Finally, we see that the LACV Gc head domain, while variable between lineages, is largely conserved at key residues at the tip of the spike. Given their location, we hypothesize that these residues may be critical for trimer formation and stability, interactions with an unknown LACV receptor or other host factors on the cell surface to facilitate entry, and/or the structure and function of the Gn glycoprotein. In the case of the Gc N609D variant, we speculate that this variant may influence trimer formation or disassociation during entry, leading to reduced infection or infection via an alternative pathway that attenuates virus dissemination and overall pathogenesis. An additional hypothesis may be that these Gc head domain residues are important for proper spike assembly, meaning that changes in glycoprotein structure can change host-pathogen interactions needed for virus entry. Specifically, the presence of distinct positively charged patches may suggest potential interactions with negatively charged glycosaminoglycans, as is the case for other arboviruses (38–40). Changes in the charge network of the Gc head domain could explain why the different lineages of LACV differ in pathogenesis (41). One explanation may be due to the changes in the Gn-Gc spike, altering the function of the spike during entry. Previous work has shown that LACV G1 and G2 can individually influence cell-specific binding (42). Therefore, it may be that changes in Gc have downstream effects on Gn for multiple step binding. Future work will be important to investigate the role of the Gc head domain in the pathogenesis of LACV and other orthobunyaviruses. We find that the OROV Gc head domain, for example, is structurally similar to that of LACV, with similar residues maintained at the Gc tip. It will be important to interrogate how the head domain of orthobunyaviruses facilitates virus dissemination and disease to better understand how these pathogens establish infections.

## MATERIALS AND METHODS

### Cells

Vero cells (CCL-81, American Type Culture Collection [ATCC]) were grown in Dulbecco’s modified Eagle medium (DMEM) supplemented with 10% newborn calf serum (NBCS). BHK-21 BSR/T7 cells (43), a gift from Dr. Steven Whitehead at the National Institutes of Health (NIH), were grown in DMEM supplemented with 10% fetal bovine serum (FBS), 1% non-essential amino acids (NEAAs), 10 mM HEPES, and 1 mg/mL geneticin added every other passage to maintain T7 selection. Human neuroblastoma cells (SH-SY5Y and CRL-2266, ATCC) were grown in a 50:50 mix of Eagle’s minimum essential medium (ATCC) and F12 medium supplemented with 10% FBS. Immortalized human myoblasts were a gift from Dr. Michael Kyba at the University of Minnesota (34). Myoblasts were grown in HAM’s/F10 Nutrient Mixture supplemented with 20% FBS, 1× Glutamax, 10 ng/mL human basic fibroblast growth factor, 40 ng/mL dexamethasone, and 100 µM beta-mercaptoethanol. All mammalian cells were maintained at 37°C with 5% CO_2_. *Aedes aegypti* cells (Aag2), a gift from Dr. Paul Turner at Yale University, were maintained in DMEM supplemented with 10% FBS, 1% NEAA, and 10 mM HEPES at 28°C with 5% CO_2_. All cells were confirmed mycoplasma free by monthly testing.

### Viruses

The LACV infectious clone system was obtained from Dr. Whitehead (33). Gc head domain mutants were generated by site-directed mutagenesis of the M segment using the primers in Table 3. All plasmids were Sanger sequenced at Plasmidsaurus to ensure there were no second-site mutations. To generate each virus, BHK-21 BSR/T7 cells were transfected with 2 µg of each of the S, M, and L plasmids using Trans-IT LT1 transfection reagent (Mirus). Twenty-four hours post-transfection, media were replaced, and cells were incubated at 37°C for 5 days. Supernatants were collected, aliquoted, and stored at −80°C. To generate working virus stocks, virus from each transfection was amplified on a monolayer of Vero cells. Viruses were collected, centrifuged at 1,200 rpm for 5 min, aliquoted, and stored at −80°C. Viral titers were quantified by plaque assay as described below.

**TABLE 3:** Primers used in this study^*a*^

LACV gc variant/primer	Forward primer	Reverse primer
Gc N609D	gatgtgtgaaa**GAC**ggtgagaaatgcagcagctc	catttctcacc**GTC**tttcacacatctgcaaaaattc
Gc S615I	gaaatgcagc**ATT**tctaattgggattttgcc	ccaattaga**AAT**gctgcatttctcacc
Gc W618R	cagctctaat**CGC**gattttgccaatgaaatgaaag	ggcaaaatc**GCG**attagagctgctgcatttctc
Gc W618L	cagctctaat**CTC**gattttgccaatgaaatgaaag	ggcaaaatc**GAG**attagagctgctgcatttctc
Gc D619G	ctctaattgg**GGC**tttgccaatgaaatgaaag	cattggcaaa**GCC**ccaattagagctgctgcatttc
Gc A621V	gggatttt**GTG**aatgaaatgaaagattattac	catttcatt**CAC**aaaatcccaattagagctgctg
Gc E623A	gattttgccaat**GCC**atgaaagattattaccccgg	ctttcat**GGC**attggcaaaatcccaattagagctg
M segment PCR primers	gtagtgtactaccaagtatag	catcatatttgaaatttgcc
S segment qPCR	ATTCTACCCGCTGACCATTG	GTGAGAGTGCCATAGCGCTG
Human GAPDH qPCR	GCAAATTTCCATGGCACCGT	GCCCCACTTGATTTTGGAGG
*Aedes* Actin qPCR	AAGGCTAACCGTGAGAAGATGAC	GATTGGGACAGTGTGGGAGAC

^*a*^ Bases in bold signify the mutated codon.

### RNA extractions and Sanger sequencing

RNA was extracted using Trizol (Thermo Fisher Scientific) and the Direct-Zol plus RNA extraction kit (Zymo Research) following the manufacturer’s instructions. cDNA was generated using the Maxima H Minus First-Strand cDNA Synthesis Kit (Thermo Fisher Scientific) and used for Phusion PCR (Thermo Fisher Scientific) with the M segment sequencing primers in Table 3. PCR amplicons were purified with the Macherey-Nagel PCR clean-up kit and Sanger sequenced at Plasmidsaurus. PCR sequences were aligned to the infectious clone reference using SnapGene (version 8.0.3).

### Plaque assay

A total of 350,000 Vero cells were seeded in 12-well plates and incubated with 10-fold dilutions of each virus for 1 h at 37°C. Agarose (0.8%) in DMEM containing 2% NBCS and 1× antibiotic/antimycotic (Gibco) was added, and cells were incubated for 72 h. Following incubation, cells were fixed with 4% formalin for 1 h; agarose plugs were removed; and cells were stained with crystal violet. Viral titers were quantified by counting the number of plaques on the lowest countable dilution. Plaque size was quantified using Image Lab (version 6.1.0, Bio-Rad).

### LACV growth curves

Vero cells (55,000 cells/well), Aag2 cells (200,000 cells/well), myoblasts (55,000 cells/well), and SH-SY5Y cells (200,000 cells/well) were seeded in poly-L-lysine-coated 24-well plates. Cells were incubated with each virus at a MOI of 0.1 for 1 h at 37°C (mammalian cells) or 28°C (insect cells). The virus was removed; cells were washed twice with phosphate-buffered saline (PBS); and complete media were added. Supernatant was collected at the indicated time points, and infectious viral titers were quantified by plaque assay as described above.

### LACV infectivity assays and immunostaining

Myoblasts (10,000 cells/well), SH-SY5Y cells (50,000 cells/well), Vero cells (10,000 cells/well), and Aag2 cells (150,000 cells/well) were seeded in black 96-well Costar clear bottom plates. Cells were washed once with PBS and incubated with each virus at an MOI of 1 for 1 h at 37°C. After incubation, media containing 20 mM NH_4_Cl were added to block virus spread. Cells were incubated for 24 h and fixed in 4% paraformaldehyde (PFA). For LACV staining, cells were then washed three times with perm/wash (BD Biosciences), incubated with 0.25% Triton for 10 min, and incubated in blocking buffer (0.2% bovine serum albumin and 0.05% saponin in PBS) for 1 h at room temperature. Cells were then incubated with a 1:2,000 dilution of primary rabbit anti-LACV antibodies (a gift from Dr. Karin Peterson at the NIH) in blocking buffer for 2 h, washed extensively with perm/wash, and incubated with a 1:10,000 dilution of secondary goat antirabbit IgG Alexa488 and 4′,6-diamidino-2-phenylindole (1:1,000 dilution) for 1 h. Cells were washed three times with perm/wash, and PBS was added. The number of infected cells was quantified on the CX7 CellInsight high-content microscope.

### LACV binding assay

For LACV binding assays, clarified viral supernatants were concentrated over a 20% sucrose cushion by ultracentrifugation at 25,000 × *g* for 4 h. Virus pellets were resuspended in DMEM containing 2% NBCS. Infectious virus titers were determined by plaque assay as described above. LACV S genome segments were quantified by extracting RNA with Trizol followed by cDNA synthesis as described above. cDNA was used for RT-qPCR using Power SYBR Green (Applied Biosciences) and the primers in Table 3. An S segment DNA standard was used to generate a standard curve to quantify the number of S genome segments.

Virus binding assays were performed by seeding Aag (200,000 cells/well), myoblasts (50,000 cells/well), and Vero cells (50,000 cells/well) into 24-well plates coated with poly-L-lysine. Cells were incubated for 1 h with media containing 20 mM NH_4_Cl and then placed on ice. Each virus was diluted to an MOI of 100 (based on RNA genomes) in DMEM containing 20 mM NH_4_Cl and incubated with cells for 20 min on ice. Cells were washed three times with cold PBS, and 250 µL of Trizol was added to each well. RNA was extracted, and cDNA was generated as described above. Relative RNA concentrations were calculated using Power SYBR Green and the S segment and GAPDH (mammalian cells) or actin (Aag2 cells) primers in Table 3. Relative increases in RNA binding over mock treatment controls were calculated using the 2^−ΔΔCt^ method against an untreated control. The amount S segment in each input RNA dilution was also quantified and used to correct for starting RNA amounts.

### Mouse infections

All animal work was completed at the NYU Grossman School of Medicine under IACUC protocol IA16-01783. For survival assays, 3-week-old wild-type C57BL/6J (Strain #000664, Jackson Laboratory) and *Ifnar1^-/-^* mice (Strain #028288; Jackson Laboratory) were infected with 20,000 or 50,000 PFU of each virus, respectively, in the left rear footpad while under anesthesia with isoflurane. Mice were weighed daily and euthanized when the weight reached less than 20% of the starting weight or exhibited neurological symptoms. All mice were euthanized after 14 days. For viral titers, mice were infected with 20,000 PFU and euthanized at 3 days post-infection. The footpad and brain were harvested in 750 µL of plaquing media containing steel beads. The tissue was homogenized using a Bullet Blender Storm Pro (Next Advance) for 5 min (brains) or 10 min (footpad) at setting 12 and clarified by centrifugation. Viral titers were quantified by plaque assay as described above.

### LACV neutralization assays

At 14 days post-infection, mice were euthanized and serum was collected. Serum was inactivated at 56°C for 30 min, and twofold dilutions were made in DMEM containing 2% NCBS. Each dilution was mixed with 5,000 PFU of WT LACV or the Gc N609D virus and incubated at 37°C for 1 h. Following incubation, the virus mix was added to Vero cells (15,000 cells/well in a black 96-well Costar clear bottom plate) and incubated for 48 h at 37°C. Cells were then fixed with 4% PFA and stained for LACV antigen, and the number of infected cells was quantified with the CX7 CellInsight High Content microscope as described above.

### Protein structures, folding calculations, and alignments

To calculate ΔΔ_fold_G and ΔΔ_bind_G, the mutant’s PDB files were generated with FoldX (44) built in YASARA software using the crystallized model of LACV Glycoprotein Gc head domain biological assembly (PDB: 6H3W). First, the WT trimer was prepared in ChimeraX by deleting water molecules and glycans (45). The impact on stability and binding of each mutation was calculated by means of ΔΔ_fold_G and ΔΔ_bind_G, employing the FoldX plugin (BuildModel and AnalyzeComplex, respectively). The positive values in ΔΔ_fold_G imply mutations that are detrimental for proper folding of the trimer, and positive values in ΔΔ_bind_G imply mutations that negatively impact inter-protomer binding. The ΔΔ_bind_G values estimate the difference in interaction energy between trimer chains between mutants and wild-type protein. ΔΔ_bind_G values were calculated according to $\Delta \Delta_{bind}G=\sum_{}^{mutant\;chains}\Delta_{bind}G-\sum_{}^{WT\;chains}\Delta_{bind}G$. Experiments were performed in triplicate.

PyMOL (version 3.1.1) was used for the structures of the LACV head domain trimer (PDB: 6H3W) and head domains of OROV (PDB: 6H3X) and BUNV (PDB: 6H3V). The PyMOL mutagenesis wizard was used to introduce mutations, and the APBS Electrostatics plugin was used for analyzing charge distribution at pH 7.4. SnapGene (version 8.0.3) was used to align all LACV M segment complete protein sequences found in NCBI Virus.

### Statistics

All data were analyzed using GraphPad Prism (version 10.4.2). Data are represented as the mean and SD. *In vitro* experiments were completed three independent times with internal duplicates where noted. *In vivo* experiments were completed at least two independent times with the number of animals found in the figure legends. *P* < 0.05 is considered statistically significant.

## Data Availability

All data are available within the article and Fig. S1.

## SUPPLEMENTAL MATERIAL

Fig. S1 jvi.00892-25-s0001.tif LACV head domain alignments.

ASM does not own the copyrights to Supplemental Material that may be linked to, or accessed through, an article. The authors have granted ASM a non-exclusive, world-wide license to publish the Supplemental Material files. Please contact the corresponding author directly for reuse.
