# Supplementary figures and images for: The La Crosse virus Gc head domain is a major determinant of infection and pathogenesis
Source: J Virol. 2025 Oct 31;99(11):e00892-25. doi: 10.1128/jvi.00892-25 (PMC12645916; doi:10.1128/jvi.00892-25)

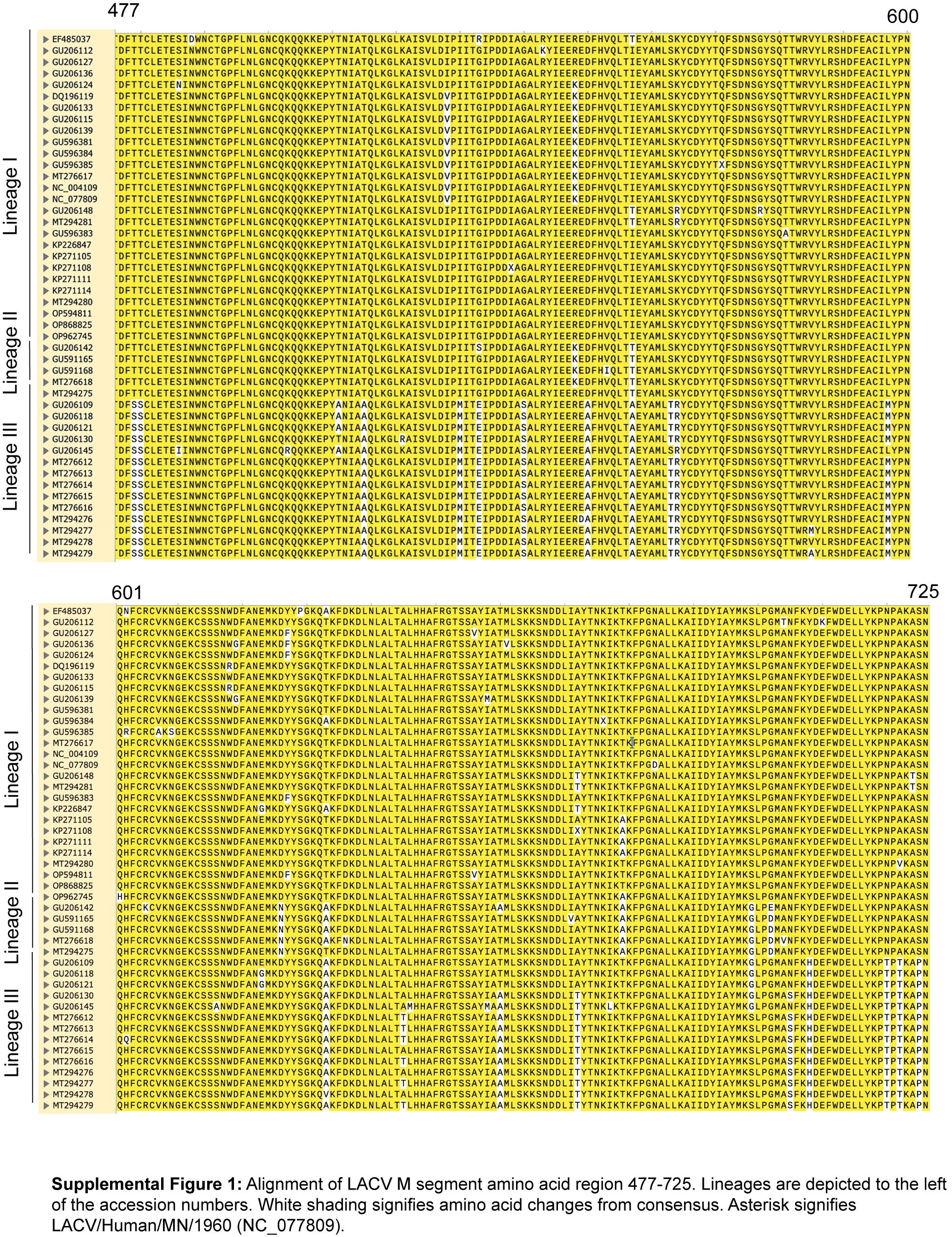

Supplement: Fig. S1 — LACV head domain alignments. [file jvi.00892-25-s0001.tif]
